# Cooperative Transition between Open and Closed Conformations in Potassium Channels

**DOI:** 10.1371/journal.pcbi.1000164

**Published:** 2008-08-29

**Authors:** Turkan Haliloglu, Nir Ben-Tal

**Affiliations:** 1 Polymer Research Center, Bogazici University, Bebek-Istanbul, Turkey; 2 Chemical Engineering Department, Bogazici University, Bebek-Istanbul, Turkey; 3 Department of Biochemistry, George S. Wise Faculty of Life Sciences, Tel Aviv University, Ramat Aviv, Israel; Harvard University, United States of America

## Abstract

Potassium (K^+^) ion channels switch between open and closed conformations. The nature of this important transition was revealed by comparing the X-ray crystal structures of the MthK channel from *Methanobacterium thermoautotrophicum*, obtained in its open conformation, and the KcsA channel from *Streptomyces lividans*, obtained in its closed conformation. We analyzed the dynamic characteristics and energetics of these homotetrameric structures in order to study the role of the intersubunit cooperativity in this transition. For this, elastic models and in silico alanine-scanning mutagenesis were used, respectively. Reassuringly, the calculations manifested motion from the open (closed) towards the closed (open) conformation. The calculations also revealed a network of dynamically and energetically coupled residues. Interestingly, the network suggests coupling between the selectivity filter and the gate, which are located at the two ends of the channel pore. Coupling between these two regions was not observed in calculations that were conducted with the monomer, which emphasizes the importance of the intersubunit interactions within the tetrameric structure for the cooperative gating behavior of the channel.

## Author Summary

Potassium channels are found, in essence, in all kingdoms of life and all types of cells, and they are involved in key biological processes. For example, they are involved in the generation and propagation of nerve impulses in the synapse and neuron. Mutations in the proteins that form the channel may lead to diseases, such as multiple sclerosis, cystic fibrosis, and cardiac arrhythmia. Because of their involvement in these and other channelopathies, i.e., channel-related diseases, they are major drug targets. The channels switch between open (ion-conducting) and closed conformations. The structural characteristics of the transition between these conformations were studied using X-ray crystallography, spectroscopic, and single-molecule techniques, as well as computations. Here we used normal-mode analysis and in silico alanine-scanning mutagenesis to understand the molecular underpinnings of this transition. Our results suggest that the transition is mediated through a network of amino acids that are coupled to each other and connect the two ends of the pore. The importance of many of these residues was noted in previous empirical studies. The calculations also suggest that interactions between subunits of the homotetrameric structure of the channel contribute to the transition. The approach may also be useful to elucidate the mechanism of other transmembrane proteins in molecular details and to suggest key amino acids that are functionally important.

## Introduction

**Figure 1:**
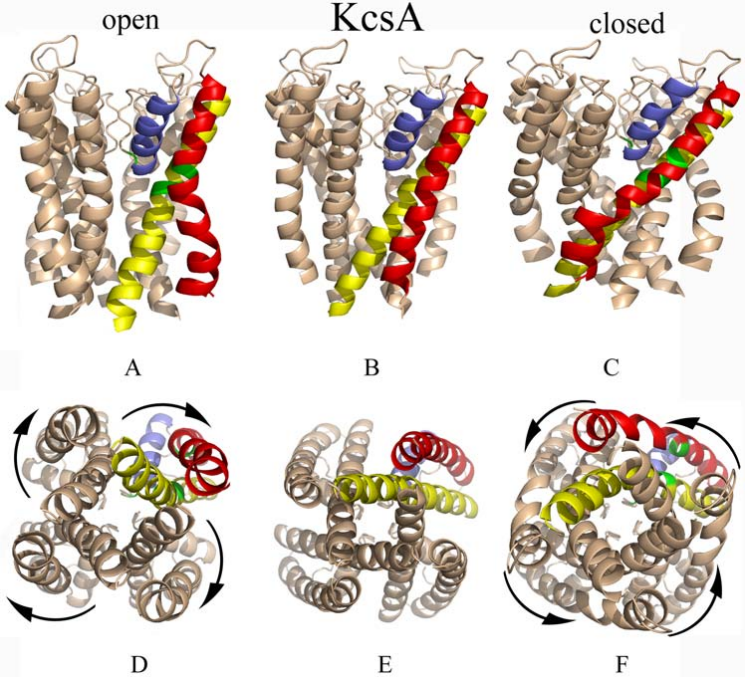
Dynamic fluctuations in the KcsA channel. The K-channel may fluctuate between the open and closed conformations. The upper panels show a side view of the X-ray crystal structure of the channel (B; PDB identifier 1bl8) and the slowest mode of fluctuations in opposite directions (A and C). The lower panels show an intracellular view of the corresponding conformations: the X-ray structure (E) and the fluctuations (D and F). The outer (M1), inner (M2), and pore helices (PH) are displayed in red, yellow and blue, respectively. The Gly99 hinge is shown in green. To open the pore, i.e., to switch from the conformation in “C” and “F” to “A” and “D,” the inner and outer helices bend around their hinge sites, the inner helices rotate in a clockwise direction and swing away from the permeation pathway. The arrows indicate the rotations of the inner helices in each subunit. The picture was prepared using Pymol (http://www.pymol.org) [64].

**Figure 2:**
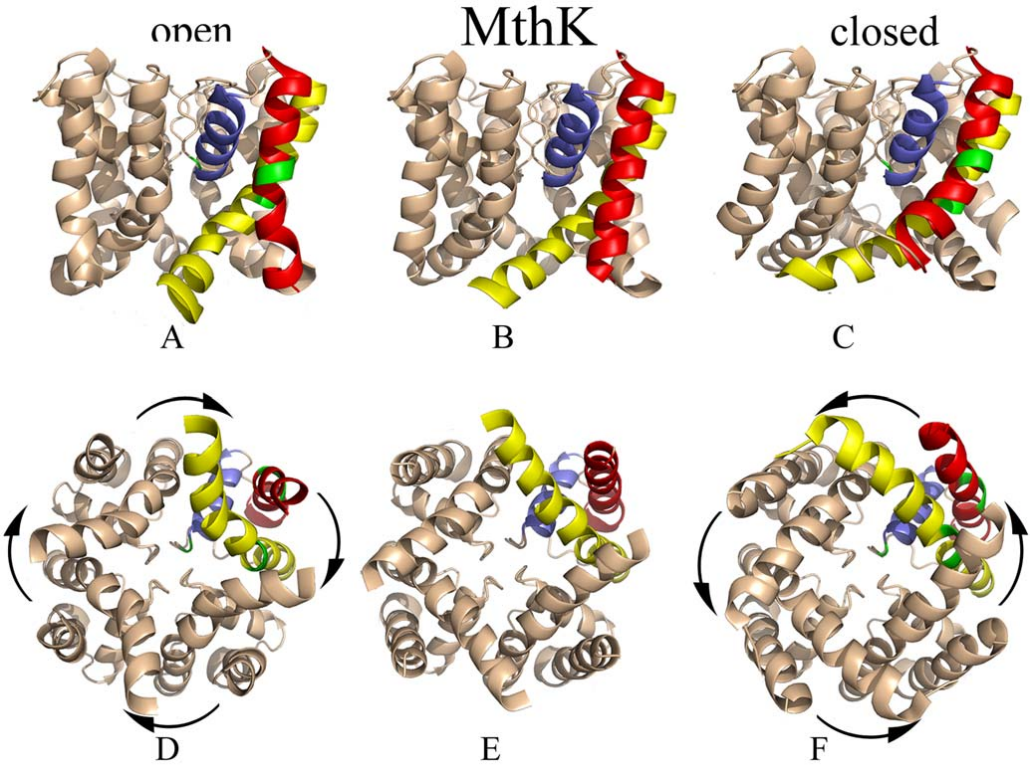
Dynamic fluctuations in the MthK channel. The second slowest mode of fluctuations of the channel is presented from a side view in the upper panels ((A) and (C)) and from an intacellular view in the lower panels ((D) and (F)). The X-ray structure of MthK (PDB identifier 1lnq) is presented in the panels in the middle ((B) and (E)). The color scheme is the same as in Figure 1. To close the pore, i.e., to switch from the open conformation of “A” and “D” to the closed conformation of “C” and “F”, the inner and outer helices bend around their hinge sites, and the inner helices rotate in a counterclockwise direction. The arrows mark the rotations of the inner helices in each subunit. The picture was prepared using Pymol [64].

Permeation and gating are two fundamental and distinct qualities of ion-channels. Permeation refers to the efficient and selective transfer of ions through the channel pore, governed by the selectivity filter, and gating refers to the control of ion access to the pore. In potassium channels (K-channels), the selectivity filter and gate are located at the opposite ends of the pore (Figures 1B and 2B).

The bacterial K-channels are very similar to each other in their 3D structure [1]–[4]. The channel is a homotetramer, where each monomer includes an outer (M1) and inner (M2) helix that are connected to each other by a pore helix (PH) and selectivity filter (Figures 1B and 2B). The selectivity filter includes the conserved TVGYG sequence motif, and is responsible for the preferential conductance of K^+^ over other ions. The segment that connects the outer and pore helices is referred to as the turret loop.

The ability of the structure to undergo conformational transitions and thus control the open/closed states of the pore in response to external signals, such as ligand binding or changes in the membrane potential, is important for proper function of the channel in the cell. Different external signals appear to bring about a similar conformational change in the pore. Studies, using EPR [5], NMR [6], X-ray crystallography [1]–[4], and single-molecule techniques [7], provided important clues about the nature of the conformational change. The structures of the KcsA channel in its closed conformation [1] and of the calcium-activated MthK channel in its open state [2] were determined. A comparison of the two structures showed that the change is associated primarily with a bend of the inner helices of the four subunits near a highly-conserved glycine residue (Figure 1B and 1E and Figure 2B and 2E for KcsA and MthK, respectively). Recently it was suggested that this motion, which leads to the transition between the open and closed state of the pore, is coupled to the selectivity filter region [6], [8]–[10], which plays a mechanistic role in channel activation/inactivation.

Systematic studies of the effects of single- and double-mutants in the channel-pore on the gating properties of the voltage-gated *Shaker* K-channel provided convincing evidence that gating is mediated through a chain of energetically-coupled residues that connect the selectivity filter to the gate [11]. This conclusion was further consolidated by computational analysis of the evolutionary history of the channels [12]: a cluster of evolutionarily-coupled residues was found, which included many amino acids that are known to be involved in gating. However, these studies failed to determine if the correlations are indicative of inter- or intrasubunit interactions, which is one of the main goals of the current work.

The understanding of molecular function in terms of structure and dynamics requires detailed knowledge of the underlying energy landscape and conformational space [13]. Perturbations, caused, e.g., by covalent modifications, mutations, or ligand binding, often change the energy-landscape and shift the sampled conformational space [14],[15]. Thus, structural changes may propagate globally in a fracture-like manner [14]. For example, binding interactions can be distributed from the binding site [16], and mutations may cause long-range structural-perturbations [17],[18]. Communication between distant sites is fundamental to the function, requiring structural elements that mediate correlated motions [19]. Identification of such correlations is a formidable effort.

The availability of X-ray crystal structures and a set of highly characterized mutational data of K-channels [1], [2], 11, 20–[22] have provided the foundation for atomistic simulation and modeling studies aiming at understanding the dynamic behavior of the channels [8], [23]–[26]. However, the study of long-time and large-scale conformational changes in the gating process is currently beyond the reach of such simulations. Normal-mode analysis based on coarse-grained potentials can fill this gap [27].

Previous studies on many different proteins demonstrated that the intrinsic slow modes of motion often correlate with the functionally-important conformational-changes [28]–[30]. This suggests that protein topology evolved in such a way that their intrinsic flexibilities ease the conformational changes required for function. Recently, Shrivastava and Bahar [31] conducted normal-mode analysis of the structures of five different K-channels. They demonstrated that the channels share similar low-frequency modes, which facilitate the opening of the pore. Also, in a very recent work [32], the opening of the KcsA pore was investigated by a combined analysis of atomistic normal-mode and Monte Carlo simulations. In the present work, which is complementary to theirs, we analyzed the KscA and MthK structures, in order to study the role of intersubunit cooperativity in K-channel gating. To this end, we combined analysis of the dynamics and energetics of the channels in a novel way.

The dynamics was investigated using two elastic-network models with simple potentials of interactions: Namely, the Gaussian Network Model (GNM) [33],[34] and the Anisotropic Network Model (ANM) [35]. These structure-based models allowed us to analyze the topologically-induced cooperative behavior of the channels in the relevant modes of motion.

In addition, a more realistic potential function, the residue-specific knowledge-based potentials [36],[37], helped us identifying a simple pairwise-coupling measure, i.e., the intensity of interactions, between the residues by in silico mutagenesis.

## Results/Discussion

We analyzed the two structures of the KcsA channel (PBD identifiers 1bl8 [1] and 1k4c [38]; Figure 1B and 1E) and the structure of the MthK channel (PDB identifier 1lnq [2]; Figure 2B and 2E). The results are presented below and in Text S1.

### Overall, Each Subunit Is Made of Four Rigid Elements, Connected by Three Hinges

We conducted calculations using two KcsA structures, 1bl8 and 1k4c, and obtained, in essence, the same results (Text S1); the results that are presented here were calculated using the former structure.

**Figure 3:**
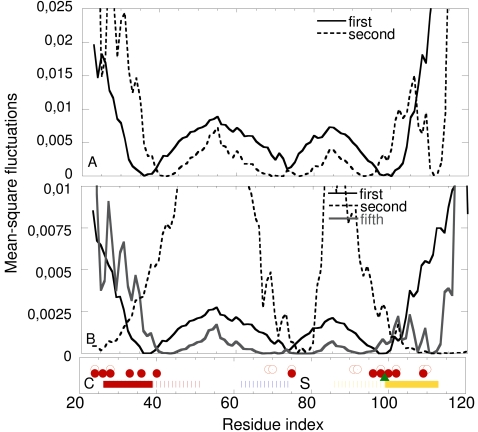
Mean-square amino acid fluctuations in the KcsA channel. The dips correspond to hinge regions. (A) Fluctuations in the first (solid) and second (dashed) slowest modes of motion of an isolated KcsA subunit. (B) Fluctuations in the first (solid black), second (dashed) and fifth (gray) slowest modes of motion of the subunit within the context of the homotetramer. (C) Structural elements and functionally important amino acids. The outer (M1), pore (PH), and inner (M2) helices are labeled in red, blue, and yellow bars, respectively, as in Figures 1 and 2. Elements and fragments of elements that are in the extracellular region of the cannel are presented using dashed bars; the bars of the inner and outer helices are dashed from the primary hinges identified in each ((A) and (B)). The approximate location of the selectivity filter is marked with “S”. The conserved Gly99 in the inner helix [2] is marked with a green triangle. Residues that are known to be functionally important based on single and double mutant experiments (Table S2 and Text S1) [8] are indicated with blank and solid red circles, respectively.

**Figure 4:**
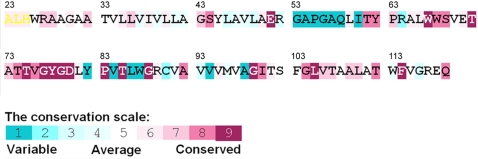
*ConSeq* evolutionary conservation analysis [61] of the KcsA channel. The *ConSeq* grades were mapped on the amino acids using the color bar with turquoise through purple marking variable through conserved amino acids.

Figure 3A and 3B display the fluctuations in the slowest modes of motion of a KscA subunit in isolation and in the tetrameric form, respectively. The first mode, which describes the most cooperative mode of motion of the protein, also dominates the average behavior of all modes of motion of the isolated and tetrameric structures (data not shown). It is evident that the first mode is, in essence, independent of the oligomerization state. This is an indication that the mode is inherent to the subunit's architecture. The curve (solid, Figure 3A and 3B) suggests that each subunit includes three hinge points: The first is around Leu36 in the outer helix, the second is around Val76 at the selectivity filter, and the third is around Gly99 in the inner helix. This is in agreement with empirical data: Leu36 (in coupling with Ser102) was noted as gating-sensitive in the correlated-mutational analysis of Yifrach and Mackinnon [11], and Val76 and Gly99 are highly conserved (Figure 4). That Gly99 is a hinge suggests an explanation for the fact that this residue is, in essence, irreplaceable [2]. Another conserved site, five amino acids downstream (residue Gly104), which is conserved as Gly or Ala (Figure 4), was also previously noted [2]. Our analysis suggested that this site is a part of the hinge around Gly99. That the Gly99 through Gly104 region functions as a hinge was also revealed by free-energy calculations [39]. Further, the flexibility around the selectivity-filter region, noted here, is in agreement with the large changes in the rotational angles of Gly77 through Asp80, suggested by NMR spectroscopy [40]. The flexibility is also in keeping with the rearrangements that were observed experimentally in the region of Leu81 through Pro83 [21]. In the latter study, this region was associated with channel inactivation.

Overall, our analysis suggested that each subunit is made of four rigid elements, connected by the three hinges. The first element (I) includes the intracellular part of the outer helix (approximately until residue 35). The second (II) includes the extracellular part of the outer helix, the pore helix and the loop that connects them (approximately residues 38–75). The third (III) includes the extracellular part of the inner helix and the loop that connects it to the selectivity filter (approximately residues 78–98). The fourth (IV) includes the intracellular segment of the inner helix (starting approximately at residue 105).

The subsequent modes correspond to more subtle and less cooperative types of motion. They show that there is another hinge close to the intracellular side of the inner helix around Ala109, which is highly conserved (Figure 4). The hinge was observed both in the isolated subunit (second mode; Figure 3A) and the tetramer (fifth mode; Figure 3B), which indicates that it is also an intrinsic property of the subunit. The presence of a hinge in this region has emerged in EPR studies [5] and in previous normal-mode calculations [41]. Interestingly, a recent normal-mode/Monte Carlo study showed that pore opening was initiated by the motion of the preceding residue, i.e., Ala108 [32].

The tetrameric organization of the subunits suppresses the amplitude of the fluctuations of the intracellular termini of the inner and outer helices (Figure 3B). The tetrameric organization of the subunits also appears to induce the emergence of a second hinge on the outer helix close to the intracellular termini (the second mode; Figure 3B, dashed curve). Interestingly, it was noted that His25 in this region of the outer helix interacts with residues at the C-terminus of the inner helices [22]. Depending on the pH, the network of interactions across the termini of the inner and outer helices stabilizes the closed state or allows the channel to open.

The hinge regions (Figure 3B, dips) correlate reasonably well with the mechanistically-informative sites of the channel, suggested by the experimental single- and double-mutations [11] (Figure 3C).

Hinge regions can also be identified based on the correlations between residue fluctuations in a given slow-mode, following the methodology described in Text S1. The hinge residues detected this way for the seven slowest modes are presented in Table S1. The majority of these residues were empirically-shown to be important for proper channel function; references to experimental studies of these residues are provided in the table.

Further analysis, conducted using the MthK structure, which corresponds to an open conformation of the channel, is presented in Text S1. The overall behavior of the MthK structure was very similar to that of KcsA, suggesting that the pore domains of K-channels share a similar gating-mechanism. The similarity between the modes of motion of these two channels has been noted already [31].

### The Open and Closed Conformations of the Channel

We conducted complementary anisotropic normal-mode analysis to display the direction of the motions predicted by the Gaussian-network model. For this, first the anisotropic normal-modes corresponding to those given by the Gaussian-network model were identified (see Text S1). The results are presented in Figures 1 and 2 for the KcsA and MthK channels, respectively. Overall, it is evident from Figure 1 that the fluctuations of the KcsA channel involve deformation towards the open conformation of the MthK structure. Similarly, Figure 2 show that MthK can deform towards the closed structure of the KcsA channel.

In the X-ray crystal structure (Figure 1B and 1E), the KscA channel is closed. Our calculations showed that fluctuations around this structure in the slowest mode may lead to two deformed conformations: one in which the pore is a bit more closed (Figure 1C and 1F) and another, in which the pore is open and would allow ion permeation (Figure 1A and 1D). Overall, the close/open transition involved motion of the rigid structural elements (I–IV) around the three flexible regions, mainly the primary hinges in the inner and outer helices (Figure 3A and 3B). The inner and outer helices bent around the hinges in residues Val37 and Gly99, respectively, and their intracellular segments (structural elements I and IV) rotated clockwise, while moving away from the permeation pathway. Such motion was suggested by Perozo [42], who argued that the fact that the motion of these two elements is coupled may help stabilizing the structure. This suggestion gained support when the structure of the MthK channel, obtained in an open conformation, came out [2]. During the conformational change, the extracellular loops of the channel rotated in the opposite direction to the rotation of the intracellular mouth of the channel. The flexibility of the pore-helix/selectivity-filter region contributed significantly to this motion. Such twisting motion around the pore-axis in the transition between the open and closed conformations was recently observed by single molecule techniques [7].

In the next anisotropic normal-mode of KcsA, the mobility of the extracellular turret loop that connects the outer and pore helices is emphasized (see Text S1). In this mode, the helix-termini are immobile, but the conformations of the pore helix and the loop between the selectivity filter and the inner helix may be affected by the mobility of the turret loop. In this context, it is interesting to note that molecular dynamics simulations and solid state NMR studies displayed conformational changes in the selectivity filter upon binding of scorpion-toxin [43].

The conformations that describe the fluctuations in the two slowest modes, discussed above also reflect the large variations around the selectivity filter, which agrees with several experimental observations [21]. Moreover, Trp67 and Glu71 were noticed here to display structural distortions. This is in keeping with empirical studies about the role of Glu71 in conformational changes and C-type inactivation, and the functional importance of Trp67 [9].

We repeated the same calculations starting from the open structure of the MthK channel and obtained very similar results (Figure 2A–F). Overall, from a cytoplasmic view, channel-opening involves a clockwise rotation of structural elements I and IV at the ends of the inner and outer helices, and closing involves counter-clockwise rotation of the same elements between the two channels. This motion is in agreement with recent single-molecule studies of the KcsA channel [7]. The channel behavior with respect to pore-opening should be a superimposition of several modes of motion, but this mode dominates the behavior (see Text S1).

The fact that similar modes of motion were obtained for two different channels in two different conformations is very reassuring. Moreover, similar calculations showed that the same pore-opening mechanism is shared also by three other channels in addition to these two [31].

### Dynamic and Energetic Couplings

We analyzed the KcsA structure, using the Gaussian network model, in search for pairs of amino acids that are dynamically-coupled. We also conducted in silico alanine-scanning mutagenesis studies in order to detect residues that are energetically-linked. One of the aspects that were explored is the cooperation between the selectivity filter and gate region at the opposite ends of the channel pore. Particular emphasis was made on the discrimination between intra- and intersubunit cooperativity within the homotetrameric structure, which is particularly challenging experimentally. Overall, there is significant overlap and complementarities between the results of the dynamic- and energetic-coupling analysis: Residue pairs, whose fluctuations are positively-correlated, are typically located in spatial proximity to each other, and are often linked on the energy landscape. In contrast, residue pairs with negatively correlated fluctuations are far from each other in space (up to tens of Angstroms) and their dynamic coupling is often mediated through chains, made of pairs of residue that are energetically coupled.

### Dynamic Coupling

The dynamic coupling was calculated as an average over the seven slowest modes, which approximates the overall behavior (see Model and Methods in Text S1). In addition we also studied the behavior of the individual modes.

#### Intrasubunit

**Figure 5:**
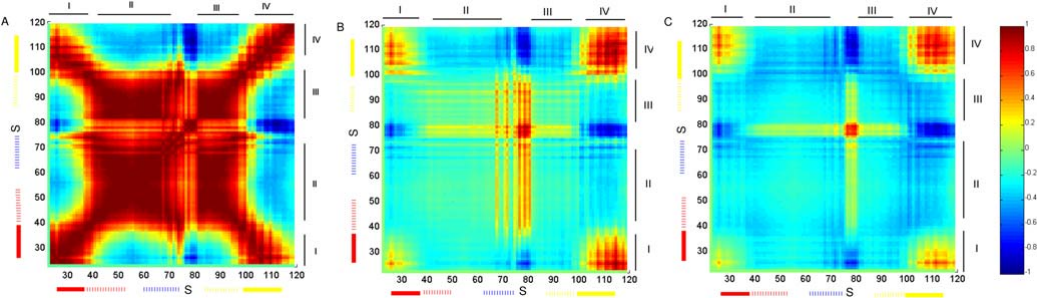
The dynamic coupling between residue pairs in the KcsA homotetramer. The axes mark the residue numbers in each subunit. The magnitude of the positive and negative correlations between the dynamic fluctuations of the amino acids (average of the seven slowest modes) is color-coded using the red-through-blue scale on the right. (A) Interactions within subunit A; (B) interactions between residues in subunit A and residues in its nearest neighbor (subunit B; the right neighbor in the homotetrameric structure from the intracellular view of Figure 1E); (C) interactions between residues in subunit A and its juxtaposed neighbor (subunit C) in the tetramer. The structural elements (I to IV) and the helices are marked on the axes using the convention of Figure 3, and the approximate location of the selectivity filter is marked with “S”. The calculations were based on the average of the seven slowest modes of motion.

The average over the slowest modes showed positive correlations between pairs of residues within each of the four structural elements, I–IV (Figure 5A). The pattern of dynamic fluctuations between the structural elements showed that the two intracellular ones (I and IV) are positively correlated with each other, which indicates that they move in the same direction. The two extracellular ones (II and III) are positively correlated with each other too but negatively correlated with the intracellular ones (I and IV). In other words, it appears as if the two intracellular elements move together in one direction while the two extracellular elements move in the opposite direction. Together with the results of the anistropic fluctuations mentioned above, this is consistent with the corkscrew-like counter-rotation of the extracellular and cytoplasmic regions of the channel, which was also observed in previous normal-mode analysis [31] and in single molecule studies [7]. According to the calculations, the motion is determined mostly by the primary hinges in the inner and outer helices (∼Gly99 and Leu36, respectively).

The motion of the inner helix, which is controlled by the primary and secondary hinges around Gly99 and Ala109, respectively, directly determines the size of the intracellular gate. In this respect, the secondary hinge may allow the minimization of the deformation in the extracellular region as the channel opens. This hinge contributes to the plasticity of the coupling between the two ends of the channel. This phenomenon was not observed in calculations of the individual subunits (data not shown), and previous normal mode analysis attributed it to the intersubunit contacts within the tetrameric structure [41]. The motion of the intracellular termini of the inner helices away from the pore at Ala108 was observed as part of the gate-opening process in recent atomistic normal-mode and Monte Carlo simulations [32].

Interestingly, in comparison to the rest of the intracellular regions (II and III), the selectivity filter displays stronger negative correlations with the ends of the inner and outer helices (regions I and IV) in the opposite end of the membrane. The selectivity filter with its flexible nature maintains this coupling, which is absent in calculations using the individual subunit. This coupling is linked to the primary (∼Gly99) and secondary (∼Ala109) hinges of the inner helix.

#### Intersubunit

The motions of the termini of the inner and outer helices of the four subunits (I and IV) are positively correlated (Figure 5B and 5C). Interestingly, the selectivity filter, located in the extracellular region, moves independently of the rest of the extracellular elements of its own subunit. However, it displays negatively-correlated fluctuations with elements I and IV, and positively-correlated fluctuations with the extracellular mouth of the other subunits. Similar interactions were detected also between the juxtaposed subunits but they are somewhat weaker (Figure 5B and 5C). The observed correlation between the motions of the selectivity filter and the gate is in line with recent EPR spectroscopy studies, which showed that these regions control channel activation and inactivation in a coupled manner [9].

Further investigation into the coupling between the selectivity filter and intracellular gate by tracing the behaviors of the individual modes is presented in the “Dynamic coupling: correlation between the selectivity filter and the gate” section in Text S1. In summary, we observed an intriguing interplay between these two regions, where the sense of the coupling changes. This change, which is attributed to the two hinges of the inner helix, may have implications for the activation and C-type inactivation behavior of the channel [8],[9].

Further analysis, conducted using the open conformation of the MthK structure, is presented in Text S1. Overall, the cooperative fluctuations of MthK were mediated mainly through the same three flexible sites, which were found in KcsA. Thus, the pattern of the network of correlated fluctuations of the open and closed states of the channel was very similar.

The isolated MthK subunit showed behavior that was identical to that of KcsA (data not shown). This is in support of the suggestion that the intersubunit contacts play a role in the formation of the two states of the channel.

### Energetic Coupling

**Figure 6:**
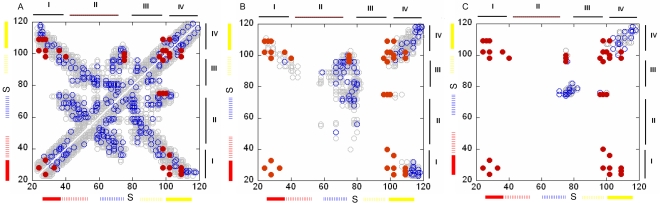
Energetic coupling. The pairwise coupling measure (CM), between residue pairs in the KcsA tetramer; within the same subunit (A), between the near neighbor subunits (B), and between juxtaposed subunits (C). The axes mark the residue numbers in each subunit. The blue circles indicate the pairs of residues that are energetically-coupled with 90% confidence based on the *z*-score analysis. The lower-bound |CM| value that specifies this confidence is 0.31*kT* (A), 0.29*kT* (B), and 0.12*kT* (C) for interresidue distance 7 Å<*r*<16.5 Å. For *r*<7 Å, the lower-bound |CM| value that specifies the confidence is 0.57*kT* (A), 0.83*kT* (B), no data (C), respectively. The gray circles mark the pairs with |CM| values between these threshold values in the respective cases and 0.1*kT*, which is determined based on the distribution of all |CM| values. The solid red circles display pairs of residues that were found to be functionally-coupled to each other in experiments (Table S1) [11]. The structural elements (I to IV) and the helices are marked on the axes using the convention of Figure 3, and the approximate location of the selectivity filter is marked with “S”.

The in silico mutagenesis analysis, presented below, reflects the intensity of the interactions. It suggests a coupling measure between the residue pairs (Figure 6A–C), and outlines key regions in the structure by identifying the residues with the highest number of contacts and/or strongest interactions. The significance of the interaction of a given residue-pair was estimated by *z*-score analysis over the distribution of all the pairs (Text S1). The interresidue interactions comprise a picture that suggests a template for the dynamic correlations as they reflect topological arrangement of the residues with a propensity for interresidue interactions. Overall, the energetically coupled residues (Figure 6A–C) displayed significant overlap with the positively correlated fluctuations (Figure 5A–C).

#### Intrasubunit

Figure 6A displays the matrix of energetic-coupling between the residue pairs within the same subunit. The diagonal regions refer to the residue pairs that are close in sequence: The primary and secondary hinge sites (Gly99 and Ala109, respectively) in the inner helix, the extended hinge region around Leu36 in the outer helix, and the pore helix showed an enhanced intensity of interactions with their near neighbors. Interactions were observed also between the two hinges of the inner helices. Off-diagonal regions refer to residue pairs that are far from each other in sequence. For example, the Gly-hinge region appeared to interact with the hinge region of the outer helix and the pore helix/selectivity filter. The extracellular ends of the transmembrane helices interacted with the adjacent extracellular loop. Additionally, the pore helix interacted with the selectivity filter; the two loops at the extracellular mouth interacted with each other; the turret loop interacted with the pore helix.

#### Intersubunit

Figure 6B displays the energetic-coupling between two nearest-neighbors subunits. The residues around the Gly99 and Ala109 hinge regions in the inner helix, the Leu36 hinge region in the outer helix, and the pore-helix/selectivity-filter region of one subunit are energetically-coupled to residues in the extended Gly99 hinge region of its near neighbor subunit's inner helix. The selectivity filter is energetically-coupled strongly with the pore helix and selectivity filter of its near neighbor. The calculations also showed energetic-coupling between the intracellular termini of the transmembrane helices of two near neighbor subunits.

Figure 6C shows energy-coupling between two juxtaposed subunits. Residues in the selectivity filters of these units were coupled to each other. Also residues near the secondary hinges (Gly109) of the inner helices were coupled.

We looked at the interaction intensities and number of residues that are energetically coupled to a given residue, and identified hotspot residues, following the criteria that are described in Methods below. The hotspots are listed in Table S1. We also repeated the calculations including only the interactions within a subunit. The difference reflects the residues that were most affected by the intersubunit interactions: The pore-helix/selectivity-filter regions and the secondary hinge region in the inner helix.

Our analysis showed that many of the hotspot and energetically-coupled residues have been reported to be important for proper function of the channel (Text S1). Some of these residues have been implicated in activation and C-type inactivation. For example, mutants of Glu71 are known to lead to two conformations, one is conducting and the other is not [9]. Glu71 is amongst our list of hotspot residues, and it shows enhanced interactions with residues in the selectivity filter. It is interesting to view this connection within the context of the dynamic-coupling between the selectivity-filter and the gate, and the flip in the sense of the couplings between these two regions. (Dynamic coupling: correlation between the selectivity filter and the intracellular gate in Text S1).

#### Comparison with experimental double-mutant cycle analysis

Yifrach and MacKinnon [11] used a double-mutant cycle analysis to detect pairs of amino acids that cooperatively affect channel-gating. A summary of their results is provided in Table S1, which correlates with our calculations in Figure 6A–C. The low resolution nature of our calculations precludes the possibility of making a one-to-one correspondence with the experimental data. However, the intensity of the interresidue interactions clustered in certain regions enables a rough comparison.

For example, residues in the selectivity filter were coupled to residues at the N- terminus of the inner helix, close to the hinge around Gly99. In our calculations, coupling was observed both within (Figure 6A) and amongst (Figure 6B and 6C) subunits. The pair of primary hinges in the inner and outer helices had higher propensities for the intra- rather than intersubunit interactions. The pairs associated with residues around the Gly99 hinge could be related to both intra- and intersubunit interactions. Interestingly, part of the cooperativity that was detected between the hinges around Gly99 and Ala109 in the inner helix seems to be more emphasized via the intersubunit interactions (Figure 6B). The other pairs associated with Ala109, i.e., Ala109 and the residues at the intracellular termini of the outer helix, were coupled mainly due to intrasubunit interactions.

#### The isolated subunit versus the subunit within the tetramer

The most significant difference in the comparison of the intramolecular correlations of an isolated subunit vs. the subunit within the context of the tetrameric structure was the absence of any correlation between the selectivity filter and the intracellular termini of the inner and outer helices in the former (data not shown). This is a key correlation that involves communication between the two ends of the permeation pathway. Also, the hinge around Ala109 in the inner helix, which is a generic hallmark of the isolated subunits of both KcsA and MthK, did not contribute to the structural fluctuations of the individual subunit (see Dynamic Couplings). This observation is mainly due to the absence of the intermolecular energetic connectivity (see Energetic Couplings).

Overall, it seems that the intersubunit contacts within the tetrameric structure may activate existing modes in a functionally related way. This is yet another example of a general principle that was found in other cases as well [27], [44]–[46].

### Cooperativity

We identified a network of cooperative fluctuations that facilitates the communication between the two openings of the pore, i.e., from the selectivity filter in one end to the gate in the other. The fluctuations of these two regions may be in the same or opposite directions, depending on the motion frequency. The determinants of this communication are mechanically-informative regions of the structure, which are subjected to intra- and intersubunit interactions. Here we relate our results on the cooperative behavior to previous investigations.

Yifrach and MacKinnon found gating-sensitive mutations both in the extra- and intracellular sides of the channel [11]. Our results agree with their findings in that we saw dynamic and energetic couplings between residues at the two ends of the permeation pathway. A hinge-lever motion could maximally amplify the fluctuations at the extracellular side by small movements at the intracellular side [41]. The calculations attributed this behavior to the hinges in the transmembrane helices, which are intrinsic to the individual subunits. On the other hand, intersubunit interactions facilitated the coupled fluctuations between the selectivity filter and gate region. The same is true for the role of the secondary-hinge around Ala109 in flipping the sense of the coupling between these two regions and reducing the deformation of the intacellular termini of the inner helices. Overall, our results provide further support to the notion that the selectivity filter is also an activation gate [8],[9],[47], as well as to the importance of the intracellular sides of the helices in the gating [17]. Further, we showed that the intersubunit contacts contribute significantly to the emergence of these functionally-important motions.

It was suggested that some structural units, such as the loops at the extracellular mouth and pore helices, allosterically control the selectivity filter [43],[48]. Our calculations supported this notion in that the selectivity filter was found to interact extensively with the extracellular mouth of the channel. Interestingly, this interaction is predominantly due to intersubunit couplings of the fluctuations (Figure 5A–C) and changes with respect to the sense of the coupling between the selectivity filter and gate region (Figure S1A–F). Strong attractive interactions of the N-terminal part of the inner helices and the pore with the C-terminus of the outer helices were pointed out in molecular simulations [26]. Our calculations suggested that this is mainly due to the strong intra-subunit correlations between the fluctuations of the respective regions. On the other hand, the coupling of the ions and the structural fluctuations of Val76 and Gly77 in the selectivity filter to the side-chains of Glu71, Asp80, and Arg89 near the extracellular side [8] could be explained by the dynamic coupling between the subunits (Figure 5B, Figure S1B, and Figure S1E).

Overall, our calculations suggested that the cooperative fluctuations in the channels are mediated via energetic-couplings between key regions in the structure. The analysis provides the means to disintegrate the observed functional behavior into its intra- and intermolecular components.

### Conclusion

The K^+^ channels are able to undergo conformational transitions in response to external forces, such as ligand binding or electrical signals, that lead to the opening or closing of the pore, i.e., the gating. Recently, Miloshevsky and Jordan, [32] used a hybrid method, based on atomistic normal-mode analysis and Monte Carlo simulations, and suggested a molecular mechanism for the opening of the KcsA channel. Their approach is based on atomistic representation (and elaborated calculations). Thus, one cannot expect perfect matching between the motion pathway that they suggest and the results obtained using coarse-grained approaches of the type that was used here. Furthermore, the normal-mode analysis suggests only projections of the real motion. Nevertheless, the channel-opening pathway that was observed in the simulations of Miloshevsky and Jordan is similar to the motion suggested in the present normal-mode analysis (Figure 1A–C) and in Shrivastava and Bahar [31], both of which are based on a single parameter. It is important to note that in contrast to the study of Miloshevsky and Jordan, our goal was not to suggest a hypothetical pathway for channel opening but rather to suggest the molecular underpinnings of the motion.

The main concern regarding our calculations, perhaps with computational analysis in general, is to determine how much of the results are biologically relevant. For example, how many of the slowest modes should be considered in order to capture the functional dynamics of the channel? The analysis of the slow modes individually, and in groups of similar frequencies, suggests their contribution to the overall dynamics and allows considering these that reflect the intrinsic behavior of the structure. Here we used two elastic network models, namely anisotropic normal-mode and Gaussian network model analyses, which complement and assure the predictions at the same time. On the other hand, we conducted calculations using two different structures of the KcsA channel and obtained, in essence, the same results. We also carried out calculation for another K-channel: MthK, and obtained similar results. In this respect, it is important to notice that KcsA and MthK share the same fold but only 25% sequence identity. They represent two states of the channel: Open and closed; and they are significantly different in the lengths of the helices and the intersubunit contacts. Thus, the fact that they share similar modes of motion is far from being trivial. It is noteworthy that Ivet Bahar's lab analyzed five different structures of potassium channels and showed convincingly that essentially these structures share the same slow modes of fluctuation (although their order of importance changes) [31].

As another mean to examine the validity of the results, we investigated the coupling between amino acids based on two complementary methods, i.e., we looked at dynamic- and energetic-coupling and analyzed this coupling with respect to the channel function. As we pointed out above, there is good correlation between our results and previous computational and experimental studies.

Our analysis provided a plausible fingerprint of the network of coupled dynamic fluctuations that may govern K-channel gating. This network revealed coupling between the selectivity filter and gate region that are located in the opposite sides of the membrane. The change in the direction of the couplings between the fluctuations of these two regions is the reminiscent of the interplay between these two regions and may refer to the different states of the channel. Each subunit includes inherent modes of motion, but gating results from cooperative effects of the tetrameric structure rather than independent motions of the individual subunits. In general, the results implied that the gating and cooperativity in this process are determined by the overall channel architecture rather than the details as reflected in the amino acid sequence.

## Materials and Methods

### Structures

Three structures of the homotetrameric channel were used: two of KcsA (PDB identifiers: 1bl8 [1] and 1k4c [38]; Figure 1B) and one of MthK (PBB: 1lnq [2]; Figure 2B). The KcsA (MthK) monomer contains 97 (80) residues, with the outer helix between residues 27–51 (22–35), the pore helix between residues 62–74 (49–58), the selectivity filter between residues 75–79 (59–63) –TVGYG, and the inner helix between residues 86–112 (71–95); 22 (18) residues are missing in the N-terminus.

### Elastic Network Models

The Gaussian Network Model (GNM) [33],[34] and its extension (Anisotropic Network Model) ANM [35] were previously described [23]. GNM predicts the relative magnitudes of the fluctuations, whereas ANM can also predict the directionalities of collective modes of motion in addition to their magnitudes. Here we used GNM to calculate the mean-square fluctuations and the correlation between the fluctuations of residues, and ANM to generate the conformations that describe the fluctuations of residues in the principal directions of motion. A more detailed explanation of the models and discussion of their limitations are provided in Text S1.

### Energetic Coupling

Site-directed mutagenesis has been a powerful tool to study interactions in native proteins and in folding intermediates in experimental [49]–[53] and computational [54]–[57] studies. Here, we used a coarse-grained in silico alanine-scanning mutagenesis to identify coupling, i.e., the intensity of interactions, between pairs of residues in the channel. We used a low-resolution chain model [58] and statistical, knowledge-based, potentials [36],[37],[59],[60], where the total interaction energy of each residue is composed of short [37],[59],[60] and long [36] range interactions.

We mutated, in silico, single residues or pairs of residues to Ala, while keeping the rest of the amino acids unaltered. For simplicity, we assumed that mutant and wild type have the same backbone and side chain conformations. Taking the wild type as a reference, we calculated the energy of the singly- and mutated protein. The absolute difference in the energy of the double mutant in comparison to that of the corresponding single mutants was taken as a measure of the degree of energetic-coupling. The larger the magnitude of the difference, the more coupled the residues were. This strategy is related to the experimental studies of Yifrach and MacKinnon [11].

The details of the potential functions employed and the definition of the coupling measure are provided in the Text S1. The description includes discussion of the limitations of method.

### The Detection of Hotspot Residues

Hotspots are defined as residues that are energetically-coupled strongly to ample residues. Here they were detected using the following criteria. First, we included only coupling that was stronger than a preset threshold that was defined based on *z*-score analysis (Text S1). Second, we required coupling to a total of seven residues. This value was determined ad hoc based on the distribution of the number of interactions per residue. A bimodal distribution was observed: most of the residues interacted with a small number of residues, and a minority of the residues interacted with 7 or more. The latter were considered to be hotspot residues.

### Evolutionary Conservation

The degree of evolutionary conservation of the amino acids was calculated using the ConSeq web-server [61] (http://conseq.bioinfo.tau.ac.il/) with default parameters. Using KcsA as input, 50 homologous sequences were collected from the SWISSPROT database [62], and the evolutionary rates of the amino acid positions were estimated using an empirical Baysian inference method [63]. The results were color-coded into the sequence using the key in Figure 4.

## Supporting Information

Text S1 Supporting Information
(0.07 MB DOC)

Figure S1 The decomposition of the dynamic couplings reflected by the fluctuations in the average of the seven slowest modes presented in Figure 5 as the average of the slowest three modes (1-through-3) (A–C) and the following next slowest four modes (4-through-7) (D–E): (A) and (D) are couplings within subunit; (B) and (E) are the couplings between the residues of two near neighbor subunits (the right neighbor in the homotetrameric structure from the intracellular view of Figure 1E); (C) and (F) are the couplings between the residues of two juxtaposed subunits. The magnitude of the positive and negative correlations between the dynamic fluctuations of the amino acids is color-coded using the red-through-blue scale on the right. The structural elements (I to IV) and the helices are marked on the axes using the convention of Figure 3.
(8.0 MB TIF)

Figure S2 Mean-square amino acid fluctuations in the MthK channel; the dips correspond to hinge regions. (A) Fluctuations in the first (solid) and second (dashed) slowest modes of motion of an isolated MthK subunit. (B) Fluctuations in the average of the first two slowest mode (dashed), third (solid) and tenth (gray) slowest modes of motion of the subunit within the context of the homotetramer. (C) Structural elements and functionally important amino acids. The outer (M1), pore (PH) and inner (M2) helices are labeled in red, blue and yellow bars, respectively, as in Figures 1 and 2 and Figure 3. Elements and fragments of elements that are in the extracellular region of the cannel are presented using dashed bars; the bars of the inner and outer helices are dashed from the primary hinges identified in each ((A) and (B)). The approximate location of the selectivity filter is marked with “S”.
(2.3 MB TIF)

Figure S3 Pore-radius profiles as a function of the distance measured along the pore centre line for the crystal and the deformed structures of KcsA and MthK, respectively: (A) The deformed structure with dashed back curve is referring to the open conformation presented in Figure 1A. The deformed structure with the solid black curve is referring to the closer conformation in Figures 1C. (B) The deformed structure with dashed back curve refers to the open conformation of Figure 2A. The deformed structure with the solid black curve refers to the closed conformation of Figure 2C.
(2.4 MB TIF)

Table S1 Functionally important amino acids.
(0.11 MB DOC)

Table S2 A list of the amino acid positions that correspond to mutations that affected the voltage-dependent gating of the Shaker K^+^ channel [11]. For single mutations, the mutations with perturbations greater than 1 kcal/mol were considered.
(0.03 MB DOC)
