# Supplementary material for: Cooperative Transition between Open and Closed Conformations in Potassium Channels
Source: PLoS Comput Biol. 2008 Aug 29;4(8):e1000164. doi: 10.1371/journal.pcbi.1000164 (PMC2528004; doi:10.1371/journal.pcbi.1000164)
Supplement: Text S1 — Supporting Information (0.07 MB DOC) [file pcbi.1000164.s001.doc]

**SUPPORTING INFORMATION**

**Model and Methods**

**Elastic Models**

**GNM.** In the GNM [33,34] the protein is modeled as a three-dimensional elastic network. The junctions are the C^α^-atoms. The interactions between the residues within a cutoff distance of 10**Å** are connected by elastic springs with a uniform force constant γ [65]. Residues i and j are assumed to undergo Gaussian fluctuations about their mean positions in the separation **R**_ij_ = |**R**_j_ - **R**_i_|, where **R**_i_ and **R**_j_ are the respective position vectors of the ith and jth C^α^-atoms.

The total potential of a protein structure in this model is given [33] by

V = (γ/2) Δ**R**^T^ **Γ**Δ**R** (1)

Where Δ**R** is N-dimensional vector of the fluctuations of Δ**R**_i_ in **R**_i_ of the individual sites, Δ**R**^T^ is its transpose, and **Γ** is the connectivity matrix (or Kirchhoff) matrix. For the special case of all pairs interacting by harmonic potential, **Γ** may be viewed as the matrix of the second derivatives of inter-residue interactions. The correlation between Δ**R**_i_ and Δ**R**_j_ is calculated [33,34] as

< Δ**R**_i_ . Δ**R**_j_> = (3k_B_T/γ) [**Γ**^-1^]_ij_ = (3k_B_T/γ) Σ_k_ [λ_k_^-1^ **u**_k_ **u**^T^_k_ ]_ij_ (2)

λ_k_ is k_th_ eigenvalue of **Γ** and is representative of the frequency of the kth mode of motion, which is ((γ λ_i_) ½). **u**_k_ is kth eigenvector, k_B_ is the Boltzmann constant and T is the absolute temperature in degrees Kelvin. This equation provides a simple means by which the dynamics can be decomposed into a series of N-1 modes for N number of interacting residues. As the contribution of each motion is scaled with the inverse frequency of that mode, the slowest modes contribute the most to the predicted fluctuations. A few slowest modes were shown to be collective and possibly relevant to the functionality of biomolecules [66]. The distribution of the eigenvalues could suggest how many of the slowest modes should be included in order to describe the cooperative dynamics at a preset accuracy.

**ANM.** In the ANM [35], **Γ** is replaced by Hessian matrix **H** of the second derivative of the intramolecular potential function in eq 1. **H** is 3N x 3N symmetric matrix and composed of N x N super elements **H**_ij_ each of size 3 x 3, given by the second derivatives of V with respect to **R**_i_ and **R**_j_ of C^α^-atoms of respective i^th^ and j^th^ residues. The correlation between Δ**R**_i_  and Δ**R**_j_ decomposed into 3N-6 modes of motions is then given by

<Δ**R**_i_ . Δ**R**_j_> = (3k_B_T/γ) tr [**H**^-1^] = (3k_B_T/γ) Σ_k_ tr[λ_k_^-1^ **u**_k_ **u**^T^_k_ ]_ij_  (3)

tr[**H**^-1^]_ij_ is the trace of the ij^th^ submatrix [**H**^-1^]_ij_ of **H**^-1^. It refers to the three different components of Δ**R**_i_  and Δ**R**_j_; whereas, when i=j, the self correlations between the components Δ**R**_i_  are obtained. Here the fluctuation vectors allows to construct and explicitly view pairs of alternative conformations sampled by the individual modes, simply by adding the fluctuation vectors ±Δ**R**_i_ to the equilibrium position vectors in the respective modes.

**The statistical potentials in energetic coupling**

The total potential energy of each residue is calculated by using the knowledge-based statistical potentials on a low-resolution chain model. It comprises both long-range non-bonded interactions and short-range conformational backbone and side chain interactions.

The long-range interaction energy was calculated by employing distance-dependent inter-residue potentials [36]. Two effective interaction sites per residue (its alpha-carbon atom and a residue-specific side-chain site) were considered, and the energy of interaction between any two interaction sites was evaluated depending on the distance in-between and the type of amino acid that the sites belong to. The total non-bonded interaction energy of i^th^ residue is found as follows

E_LR_(Φ)_i_ = Σ_j=1,4N_ E_SS_ (r_ij_) + Σ_j=1,4N_ E_SB_ (r_ij_) + Σ_j=1,4N_ E_BB_ (r_ij_) (4)

where r_ij_ is the distance between sites i and j in conformation Φ. N is the number of residues in a subunit. The terms account for potentials between side-chain sites (SS); side-chain and backbone sites (SB) and two backbone sites (BB) of residues i and j, where │i-j│≥ 3, 4, 5, respectively.

To evaluate the conformational energy of the backbone, the statistical potentials given [37] for bond angle and bond torsions are used as

E_SR_(Φ)_i_ = E(θ_i_) + [ E(φ_i_^-^)/2 + E(φ_i_^+^)/2 + ΔE (φ_i_^-^, φ_i_^+^)] + [ΔE (θ_i_, φ_i_^-^)] + ΔE (θ_i_, φ_i_^+^)] (5)

Here, the first term is to account for the bending of backbone bond angles; following terms are for the torsion of bonds φ_i_^-^ and φ_i_^+^ referring to the rotational angles of the virtual backbone bonds preceding and succeeding the i^th^ α-carbon, respectively, and for the pairwise interdependence of the torsion and/or bond angle bending.

For the side chains, the statistical potentials based on the distributions for packing of side chains in low-resolution models [59] are used as

E^s^_SR_(Φ)_i_ = E(l_i_^s^) + E (θ_i_^s^) + E (φ_i_^s^) (6)

where l_i_^s^, θ_i_^s^ and φ_i_^s^ are the bond length, bond angle and torsion angle of side chain i^th^ residue.

The so-called pairwise coupling measure, CM, between the residues of the pair is calculated as

CM= ΔE_ij_-ΔE_wild_ – ΔE_i_-ΔE_wild_ – ΔE_j_-ΔE_wild_  (7)

Here ΔE_ij_, ΔE_i_ and ΔE_j_ refer to energies of the structure in double mutations of i and j and single mutations of i and j, respectively. E_wild_ is the energy of the wild type structure. CM with this definition may serve as a measure of connectivity strength between residues. Statistical analysis, based on z-score, was used to assign significance to the calculated coupling values.

**A z-score analysis of the energetic coupling**

First, the energetic coupling values were considered separately for intra-subunit and inter-subunits interactions. Secondly, in each type of interactions, intra- or inter-subunit, the pairs were also clustered into two groups: the pairs that are separated by a distance of r < 7 and 7 Å < r < 16.5 Å. The cutoff value of 7 Å corresponds to the width of the first coordination shell [36]. 16.5 Å was selected based on the existence of any interacting residue-pair with a nonzero coupling-value. Prior to z-score calculations, the clustering of the residue pairs based on the inter-residue distances allowed accounting for the differences in the coupling values’ profiles with the inter-residue distances. This is to identify relatively more pronounced interactions in both groups of the residues pairs.

In the z-score calculations, the mean and variance of the coupling values were calculated. Then, the threshold value was chosen such that any pair that displays a coupling above it is energetically-coupled with at least 90% confidence (i.e., with probability P > 0.9).

The residue pairs that are presented with the blue circles in Figure 6 are those above these thresholds. The gray circles are the residue-pairs with the correlation values between the threshold estimated by the z-score calculations and 0.01 kT. The lower bound 0.01 kT was estimated by looking at the distribution of the coupling values for all pairs of residues. There is a steep change around this value and any pair with a coupling value of > 0.01 kT was plotted in gray circle.

**Results**

**Identification of hinge regions**

The minima of the slowest mode curves suggest the hinge regions in a structure (Figure 3). Alternatively, the hinge residues could also be detected through the analysis of cross- correlations of the residue fluctuations. In a single mode, the change in the sign of the correlation values between "–" and "+" suggests a flexible joint that connects the rigid structural units. This analysis was carried out for the seven slowest modes incorporated in our fluctuation analysis (Figures 3, 5 and S1). We observed the same residues that were deduced from the analysis of the slowest mode shapes (Figure 3) and a few more short flexible segments, which were also noted as functional. For example; rapid conformational changes near the C-termini of the inner helices in coupling with the selectivity filter were suggested by a recent NMR study [Baker, Nat 2007]; that agrees the enhanced flexibility of this region observed by the present calculations. A complete list of the hinge residues is provided in Table S.1.

The conformations that describe the motion in the two slowest modes by ANM were further analyzed to reflect the distortions in the rotations of the backbone bonds to accommodate the fluctuations observed in these modes. For this, each rotation is defined by the alpha-carbon positions of four successive residues and it is assigned to the third residue. Thus, each putative rotation involves also the neighbor residues. The residues with highly distorted rotations from the average structure were listed in Table S.1.

**Dynamic coupling: correlation between the selectivity filter and the intracellular gate**

The role of the selectivity filter in activation and C-type inactivation, i.e. inactivation due to a conformational change into a non-conducting state of the K-channels, has been the focus of recent studies [8-10,21,48]. The channels are inactivated within hundredths of millisecond after channel activation, and inactivation is accompanied by activation [21,67]. Thus, gating should be a complex phenomenon that involves several structural units operating cooperatively and possibly at various time scales [8]. The flexibility observed in the selectivity filter region (Table S.1) may reflect the range of conformations that are accessible to this region during gating.

The dynamic couplings of Figures 5A, 5B and 5C show the average fluctuations in the seven slowest modes, which approximates the overall behavior. They manifest the existence of negative correlations between the fluctuations of the selectivity filters and gate region within- and between- the subunits. We analyzed each of these modes separately in order to further our understanding on this.

On average, the three slowest modes show negative cooperativity between the residue fluctuations in the selectivity filter and gate region (Figures S1.A-C). And in the following four modes (4-through-7), there is plasticity in the coupling of the fluctuations of the selectivity filter with the gate region: The selectivity filter displays positively correlated fluctuations between Gly99 and Ala109 of the inner helix, and mostly uncorrelated fluctuations between Ala109 and the C terminus (Figures S1.D-F).

The analysis revealed more inter-subunit cooperative fluctuations in the channel, but the sense changed from mode to mode. For example, the extracellular mouth regions of the near neighbor subunits were positively correlated with each other in the three slowest modes (Figures S1.B), but negatively correlated in the next group of slow modes (4-through-7; Figure S1.E). A change from positive to negative correlation was also observed between the extracellular mouth regions of two juxtaposed subunits (Figures S1.C, S1.F). Regarding the interplay between the selectivity filter and the extracellular mouth region of the other subunits, the sense of the correlated fluctuations switched from positive (Figures S1.B, S1.C) to negative (Figures S1.E, S1.F). Lastly, the sense of the correlation of the gate (IV) with respect to the extracellular mouth (except the selectivity filter) changed mostly from negative to zero for the region of Gly99-Ala109 and to positive for the region of Ala109-C terminus for near neighbor subunits (Figures S1.B, S1.E). In that, the flexibility of Ala109 has a significant role. It is important to note that recent NMR studies suggested the existence of at least two conformational states, involving changes in the selectivity filter and near the C-terminal ends of the inner helices [6].

The switch in the direction of the coupling between the selectivity filter and gate regions may be a reminiscence of the two different states of the channel. These two states differ in their motion frequency and time scale. Here, the conductive and nonconductive states may be associated with the two opposing dynamic behaviors of the channel. In the 4-through-7 modes the coupling between the two mouths of the channel is weak. Since the intracellular gate in KcsA does not undergo any substantial changes during inactivation [10] we suggest that the dynamics of these modes might be related to the inactivation process.

Interestingly, similar analysis, carried out for MthK, yielded also the switch in the sense of the couplings between the selectivity filter and intracellular gate, depending on the frequency range of the modes. To see the similar behavior in both channels strengthen the importance of these couplings for a plausible functional state of K-channels in general.

**Fluctuations of the open structure, MthK**

**Auto-correlations:** We also conducted similar analysis using MthK, a homologous K-channel to the KcsA, the structure of which corresponds to an open conformation. GNM calculations were carried out for the isolated subunit and within the context of the tetrameric structure. The results for the isolated subunit are essentially identical to the results that were obtained for KcsA; the shapes of the two slowest modes are the same as those of KcsA with the hinge sites at the corresponding positions. The results for the MthK and KcsA subunits within the context of their tetrameric structures are also very similar, yet the order of the modes as well as the relative contribution of the hinges in a given mode changed. For example, the third mode in MthK corresponds to the first mode in KcsA and its first two modes are degenerative modes. These are basically the same modes with equal frequencies; the contribution of the hinge at the selectivity filter to the cooperative motion is stronger than the primary hinges at the inner and outer helices in the first (and second) mode of MthK (Figure 2S). Overall, the average behavior was preserved, and the location of the three minima in the average mode is in agreement with the experimental observations in MthK [2].

**Cross-fluctuations*:*** The cooperative fluctuations in MthK are mediated mainly through the three hinge sites, found also in KcsA. The decrease in the number of contacts between the intracellular termini of the inner and outer helices in the open state may weaken some of the correlations and at same time may enhance some of the correlations. Yet, the overall dynamics picture is preserved.

*Intra-subunit:* The pattern of correlation between the fluctuations of residues in MthK was very similar to the one observed in KcsA, excluding the undermined negative correlation between the selectivity filter and the gate. Also, the correlations between the intracellular termini of the inner and outer helices near the intracellular region were missing. This is presumably since the helices straightened out and the number of contacts between them was smaller in the open conformation of MthK in comparison to that of KcsA [2].

*Inter-subunit:* The network of the correlated fluctuations between the MthK subunits resembled that in the KcsA structure. However, the correlations among the intracellular termini of the helices were weaker and the selectivity filter correlated with more extended region of the extracellular mouth of the near neighbor subunits. The coupling between the selectivity filter and the gate was weaker but non-negligible, and it extended towards the hinge regions in both helices. In the elaboration of the slow modes within the context of the cross-correlations, MthK also displays the switch in the sense of the coupling between the selectivity filter and the gate regions, and also in other associated-regions (data is not shown).

The cooperative fluctuations of the MthK and KcsA in the isolated subunits are exactly the same (data not shown); this assures the role of the inter-subunit energetic coupling in the observed differences between the cooperative fluctuations of the tetrameric structures.

**Mapping of GNM and ANM modes**

In the present analysis, the dynamic modes of the GNM and ANM were compared to map the similar modes by correlating the mean-square fluctuations. The mode shapes in GNM were taken as a reference and the corresponding ANM modes were used to extract the direction of the fluctuations.

Comparison of the slowest mode shapes by ANM of the two channels displayed that the slowest mode, which describes the open/close transition with the hinge motion in transmembrane helices, is the slowest first in KcsA and the slowest second in MthK. The shape of this mode is the equivalent of the first mode of KcsA by GNM (Figures 3A and 3B) and the third slowest mode of MthK (Figure S3; see Fluctuations of the open structure, Mthk in *Supporting Information*).

**Comparison of the fluctuations of the two crystal structures of KcsA**

The comparison of the fluctuations by GNM and ANM of the two crystal structures, 1k4c and 1bl8, of KcsA yields the following results.

**GNM:** We analyzed the seven slowest mode shapes of these two structures. The comparison yielded the same mode shapes presented for 1bl8 in Figure 3A and Figure 3B The only difference was that the fifth mode shape of 1bl8 corresponded to the seventh mode shape of 1k4c, where we observe the second hinge in the inner helix. Nevertheless, the analysis of the cross-correlation of the seven slowest modes yielded very similar correlation pattern between the two structures.

**ANM**: The types of motion in the six slowest modes by ANM for the two structures are basically the same. The only difference is regarding a degenerative mode, which appears as the second-and-third modes in 1k4c and as the third-and-fourth modes in 1bl8. In this case, the fourth mode of 1k4c is the same as the second mode of 1bl8. The fifth and sixth modes are the same between 1k4c and 1bl8.

**Hotspot residues by energetic coupling**

The energetically susceptible residues suggested here correlated with the experimental findings (Table S1). For example; residues Glu51 and Glu71. Glu51 was suggested to be associated with C-type inactivation [11,68] and involved in the conformational twisting of residues Leu81-Pro83, which are also involved in C-type inactivation [9, 21]. Here Glu51 was also noticed to interact with Leu81 through residues Leu59 and Arg64. Arg64 was also previously noted as a functional residue [9]. In this respect it is noteworthy that all these residues (i.e., Leu59, Arg64, Leu81 and Pro83) are among the hotspots that we detected.

A recent EPR study showed that mutants of Glu71 may lead to two different conformations, one is conducting and the other is not [9]. Here, Glu71 was found to interact directly with Arg64, and with Val76, Tyr78 and Asp80 of the selectivity filter. Glu71 was also noted as a hinge, which further complements its importance (Table S1).

The Trp67 and Ser69 residues were also identified here as hotspot residues, which is in line with previous studies. Ser69 was pointed out as one of the gating sensitive residues [8]. Trp67, which is known to be functionally-important [9], was observed here as intimately connected with Val70, Tyr78 and Asp80. Experimental studies suggested that Trp68, Tyr78 and Asp80 are involved in C-type activation [21]. Here Asp80, Leu81, and Ty82 were identified as hotspot residues; Tyr82 was also pointed out as stabilizing the inactivation state [9]. Tyr82 is also in the list of flexibility residues suggested by our anisotropic network model analysis (Table S1).

Additionally, His25 and several residues at the C-termini of the inner helices appeared as hotspot residues. This is also in apparent agreement with empirical data: His25 is one of the key residues that interact with the termini of the inner helices that stabilizes the closed state or allows the pore to open, depending on the pH [22]. At the C-termini of the inner helices, the residues Ph114 and Gly116 were noted [6] to display pH dependent chemical shifts changes together with some residues at the selectivity filter. Here, additionally, the calculations suggested the enhanced interactions of Phe114 with the residues at C termini of the outer.

Most of these residues involved considerable inter-subunit interactions in our analysis. Additionally, residue Leu59 was noted here as energetically important. Leu59 interacts directly with residue Leu81 of the selectivity filter, as well as with residue Glu51, which is evolutionarily conserved (Figure 4) and known to be involved in C-type inactivation. Since many of the hotspot residues are functionally-important, it would be interesting to conduct experiments to examine the importance of this residue too.

Here, it is also interesting to note that Met96 is the residue with the highest number interactions (18). Glu71; 13 interactions, and Leu59; 12 interactions, follow.

**The open/close transition of the gate: KcsA vs MthK**

The analysis of the anisotropic normal-modes of KcsA and MthK displayed that both share a similar mode where it is possible to visualize the transition between the open and closed conformations. HOLE [69] analysis of the profiles of the pore radii of these states demonstrate the transition: The two closed states of KcsA and MthK exhibited a narrow opening in the gate region while the corresponding open states of these channel manifested wide opening of this region (Figures S3.A and S3.B).

In the next slowest anisotropic normal-mode of the KcsA channel, the motion is mainly dominated by the extracellular turret loops that connect the outer and pore helices. In this mode, the helix-termini are immobile. The motion of the turret loop affects the conformations of the pore helix and the rest of the extracellular regions of the channel. This may lead to fluctuations in the size and shape of the central cavity, known to play a role in stabilizing water molecules and cations [1,70]. The flexibility of the selectivity filter region is also of importance here. There are toxin-binding sites on the turret loops. The bound toxins affect channel activity by plugging into the external pore entryway. It was suggested that it extends diagonally across the pore-mouth from one turret to another [71]. A recent solid-state NMR study revealed the importance of the intrinsic flexibility of the selectivity filter in the toxin-channel interaction [40]. Also, recent molecular dynamics simulations, validated by solid state NMR studies, displayed the conformational changes in the selectivity filter upon toxin-binding [43].

The deformed conformations reflect the distortions in the rotations of the backbone to accommodate the fluctuations observed in these modes (Table S1). Here, again, we observed that the selectivity filter, the C-terminus and some other residues at the extracellular mouth experienced noticeable distortions in addition to the hinge regions in the transmembrane helices. Trp67 and Glu71, which exhibited structural distortions, were also noted as energetic hotspots and they interacted strongly with the selectivity filter region. The involvement of Glu71 in structural distortions is in line with recent x-ray crystal studies from the Perozo Lab [9].

**References**

65. Tirion MM (1996) [Large amplitude elastic motions in proteins from a single-parameter, atomic analysis](http://prola.aps.org/abstract/PRL/v77/i9/p1905_1). Phys Rev Lett 77: 1905-1908.

66. Rader AJ, Chennubhotla, C, Yang, L-W, Bahar, I (2006) "The Gaussian Network Model: Theory and Applications" in "Normal Mode Analysis. Theory and Applications to Biological and Chemical Systems" Eds Qiang Cui and I Bahar, Chapman & Hall / CRC Mathematical and Computational Biology Series, CRC Press, Taylor & Francis Group, 41-64.

67. Gao L, Paajanen V, Wang K, Fan Z (2005) **Activation-coupled inactivation in the bacterial potassium channel KcsA.** Proc Natl Acad Sci U S A 102: 17630-17635.

68. Larsson HP, Elinder F (2000) A conserved glutamate is important for slow inactivation in K+ channels. Neuron 27: 573-583.

69. Smart OS, Goodfellow JM, and Wallace BA (1993) The pore dimensions of gramicidin. Biophys J 65:2455-2460.

70. Roux B, MacKinnon R (1999) The cavity and pore helices in the KcsA K^+^ channel: electrostatic stabilization of monovalent cations. Science 285: 100-102.

71. Armstrong C (1998) NEUROSCIENCE: The Vision of the Pore. Science 280: 56-57.

**Figures**

**Figure S1.** The decomposition of the dynamic couplings reflected by the fluctuations in the average of the seven slowest modes presented in Figure 5 as the average of the slowest three modes (1-through-3) (A-C) and the following next slowest four modes (4-through-7) (D-E): (A) and (D) are couplings within subunit; (B) and (E) are the couplings between the residues of two near neighbor subunits (the right neighbor in the homotetrameric structure from the intracellular view of Figure 1E); (C) and (F) are the couplings between the residues of two juxtaposed subunits. The magnitude of the positive and negative correlations between the dynamic fluctuations of the amino acids is color-coded using the red-through-blue scale on the right. The structural elements (I to IV) and the helices are marked on the axes using the convention of Figure 3.

**Figure S2**. Mean-square amino acid fluctuations in the MthK channel; the dips correspond to hinge regions. (A) Fluctuations in the first (solid) and second (dashed) slowest modes of motion of an isolated MthK subunit. (B) Fluctuations in the average of the first two slowest mode (dashed), third (solid) and tenth (gray) slowest modes of motion of the subunit within the context of the homotetramer. (C) Structural elements and functionally important amino acids. The outer (M1), pore (PH) and inner (M2) helices are labeled in red, blue and yellow bars, respectively, as in Figures 1 and 2 and Figure 3. Elements and fragments of elements that are in the extracellular region of the cannel are presented using dashed bars; the bars of the inner and outer helices are dashed from the primary hinges identified in each ((A) and (B)). The approximate location of the selectivity filter is marked with "S".

**Figure S3.** Pore-radius profiles [69] as a function of the distance measured along the pore centre line for the crystal and the deformed structures of KcsA and MthK, respectively: A) The deformed structure with dashed back curve is refering to the open conformation presented in Figure 1A. The deformed structure with the solid black curve is refering to the closer conformation in Figures 1C. B) The deformed structure with dashed back curve refers to the open conformation of Figure 2A. The deformed structure with the solid black curve refers to the closed conformation of Figure 2C.
