# Supplementary figures and images for: Cooperative Transition between Open and Closed Conformations in Potassium Channels
Source: PLoS Comput Biol. 2008 Aug 29;4(8):e1000164. doi: 10.1371/journal.pcbi.1000164 (PMC2528004; doi:10.1371/journal.pcbi.1000164)

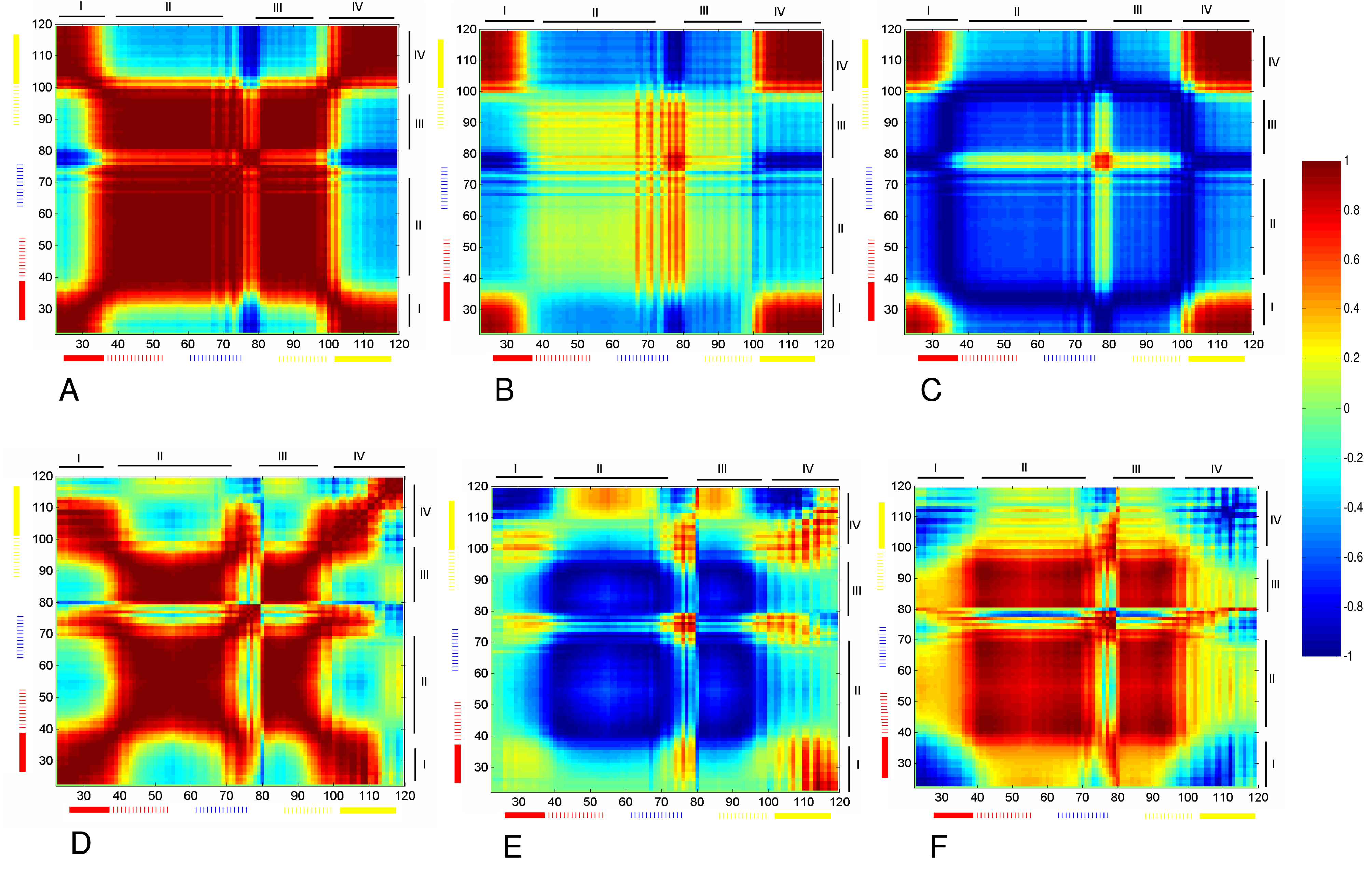

Supplement: Figure S1 — The decomposition of the dynamic couplings reflected by the fluctuations in the average of the seven slowest modes presented in Figure 5 as the average of the slowest three modes (1-through-3) (A–C) and the following next slowest four modes (4-through-7) (D–E): (A) and (D) are couplings within subunit; (B) and (E) are the couplings between the residues of two near neighbor subunits (the right neighbor in the homotetrameric structure from the intracellular view of Figure 1E); (C) and (F) are the couplings between the residues of two juxtaposed subunits. The magnitude of the positive and negative correlations between the dynamic fluctuations of the amino acids is color-coded using the red-through-blue scale on the right. The structural elements (I to IV) and the helices are marked on the axes using the convention of Figure 3. (8.0 MB TIF) [file pcbi.1000164.s002.tif]

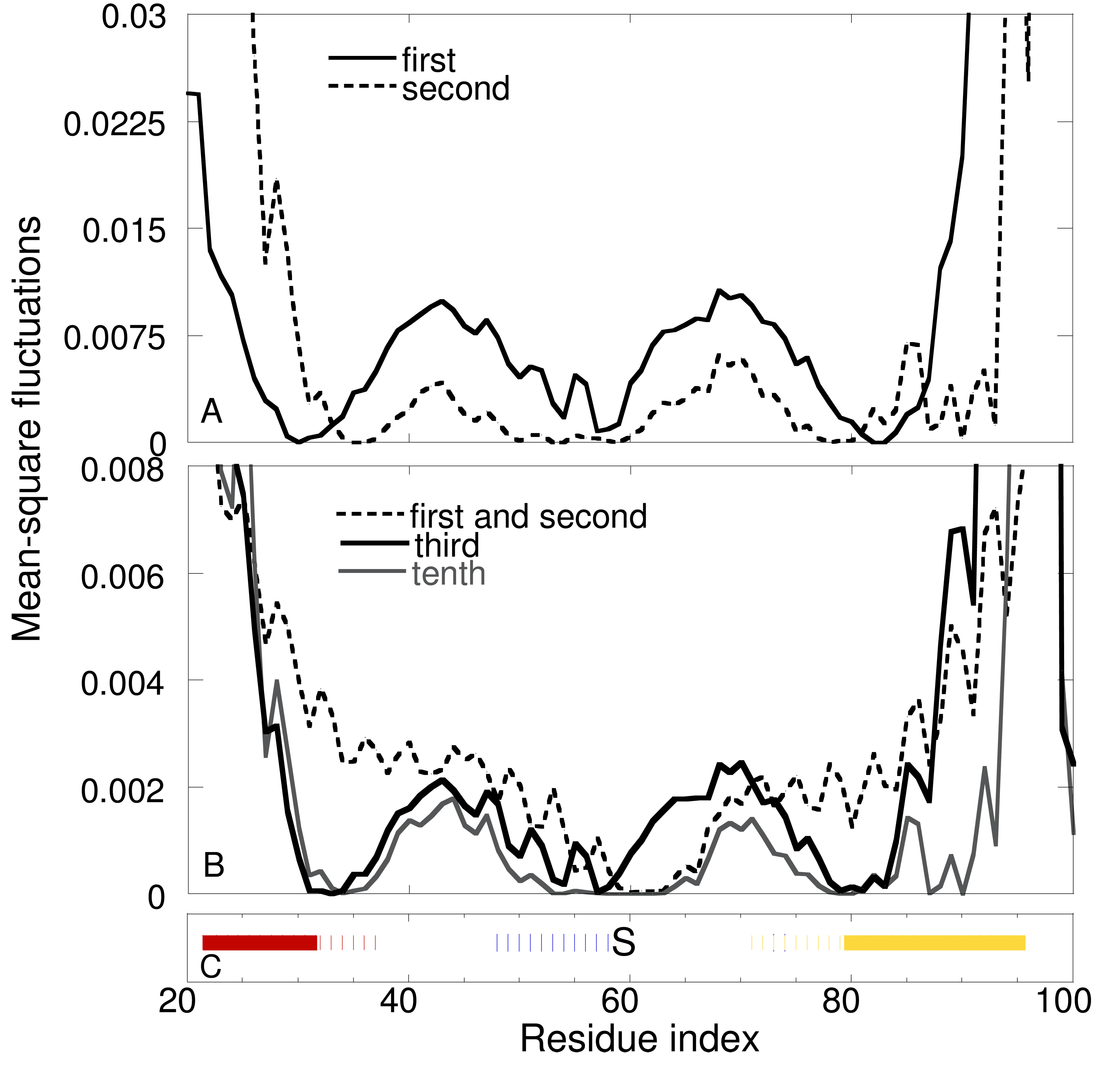

Supplement: Figure S2 — Mean-square amino acid fluctuations in the MthK channel; the dips correspond to hinge regions. (A) Fluctuations in the first (solid) and second (dashed) slowest modes of motion of an isolated MthK subunit. (B) Fluctuations in the average of the first two slowest mode (dashed), third (solid) and tenth (gray) slowest modes of motion of the subunit within the context of the homotetramer. (C) Structural elements and functionally important amino acids. The outer (M1), pore (PH) and inner (M2) helices are labeled in red, blue and yellow bars, respectively, as in Figures 1 and 2 and Figure 3. Elements and fragments of elements that are in the extracellular region of the cannel are presented using dashed bars; the bars of the inner and outer helices are dashed from the primary hinges identified in each ((A) and (B)). The approximate location of the selectivity filter is marked with “S”. (2.3 MB TIF) [file pcbi.1000164.s003.tif]

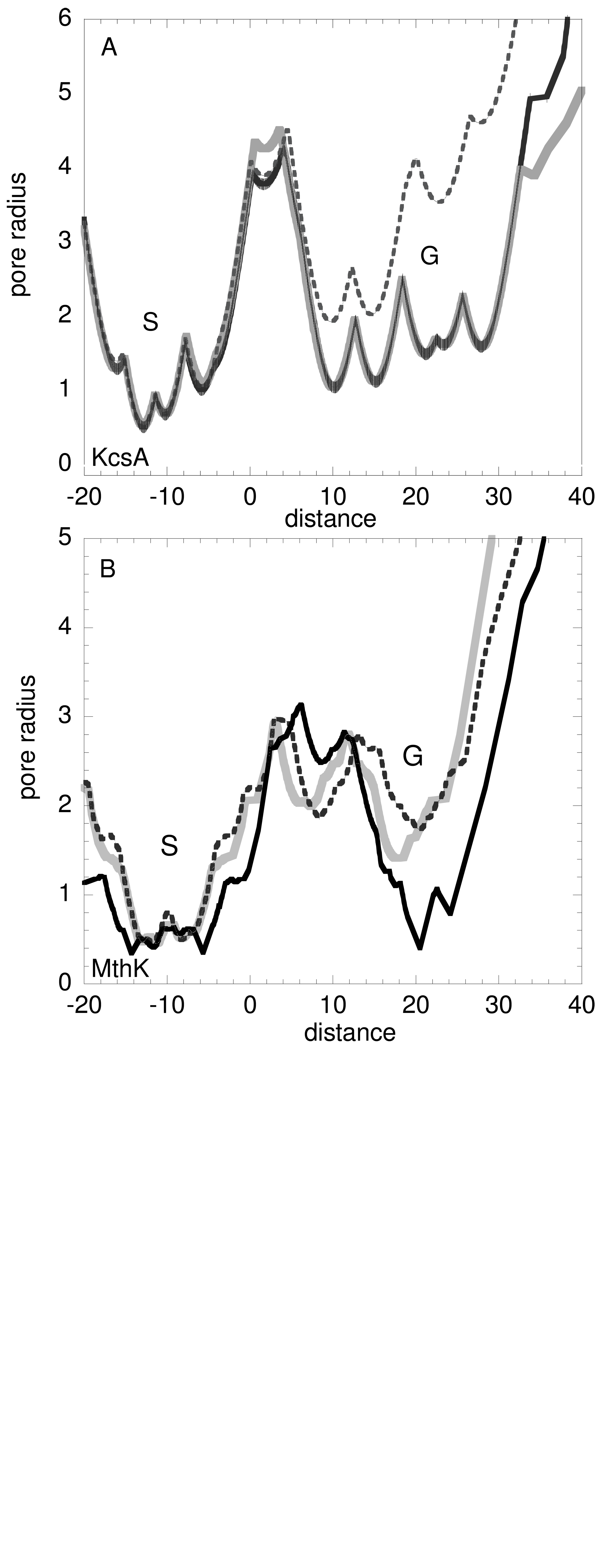

Supplement: Figure S3 — Pore-radius profiles as a function of the distance measured along the pore centre line for the crystal and the deformed structures of KcsA and MthK, respectively: (A) The deformed structure with dashed back curve is referring to the open conformation presented in Figure 1A. The deformed structure with the solid black curve is referring to the closer conformation in Figures 1C. (B) The deformed structure with dashed back curve refers to the open conformation of Figure 2A. The deformed structure with the solid black curve refers to the closed conformation of Figure 2C. (2.4 MB TIF) [file pcbi.1000164.s004.tif]
