# Supplementary material for: Cooperative Transition between Open and Closed Conformations in Potassium Channels
Source: PLoS Comput Biol. 2008 Aug 29;4(8):e1000164. doi: 10.1371/journal.pcbi.1000164 (PMC2528004; doi:10.1371/journal.pcbi.1000164)
Supplement: Table S1 — Functionally important amino acids. (0.11 MB DOC) [file pcbi.1000164.s005.doc]

**TABLE S1.** Functionally important amino acids.

| Residues | ^a^GNM | ^b^ANM | ^c^Energ. analysis  based on z-score | ^d^ConSeq  score | ^e^Exp. Observations |
| --- | --- | --- | --- | --- | --- |
| His25 |  |  | x | 6* | Interacting with C-termini of inner helices and has role in gating [22] |
| Ala32 |  | x |  | 7 |  |
| Val34 |  | x |  | 5 |  |
| Leu36 | x | x | x | 5 | Gating sensitive double mutations (with Ser102) [11]. |
| Val37 | x | x |  | 4 |  |
| Leu40 |  |  |  | 5 | Gating sensitive double mutations (with Ala98) [11]. |
| Gly43 | x |  |  | 6 |  |
| Ser44 | x |  |  | 7 |  |
| Glu51 |  |  | x | 9 | C-type inact. [11], [68] in Shaker. Interacts with Val84 and stabilizes the C-type inactivation, breakage leads to the inactivation [68]. |
| Ala57 |  | x |  | 1 |  |
| Leu59 |  |  | x | 5 |  |
| Arg64 |  |  | x | 3 | Affect gating [9]. |
| Trp67 | x | x | x | 9 | Two conformers exist. Participates in C-type inactivation, activation [9]. |
| Trp68 | x |  | x | 6 | Interacts with Asp80 in C-type inactivation [68].  Gating sensitive single mutations at Ser69 and Val70 [11]. |
| Ser69 |  |  | x | 8 | Gating sensitive single mutation [11]. |
| Glu71 | x | x | x | 7 | Mutant increases the open probability [21].  Mutants are gating sensitive, associated with C-type inactivation [9]. Influence gating [6] |
| Thr72 |  |  | x | 9 |  |
| Thr74 |  |  | x | 8 |  |
| Thr75 |  |  | x | 9 | Gating sensitive single and double mutations at Thr75 (Met96, Gly98, Ile100) 11]. |
| Val76 | x |  |  | 8 | Gating sensitive single and double mutations at Thr75 (Met96, Gly98, Ile100) [11]. |
| Gly77 | x |  |  | 9 | Large changes in backbone angles by NMR [40]. |
| Tyr78 |  |  | x | 9 | Large changes in backbone angles by NMR [40].  Inter-subunit cooperativity in C-type interactions [68].  Coupling with C-termini of helices [6] |
| Gly79 | x | x |  | 9 | Large changes in backbone angles by NMR [40]. Coupling with C-termini of helices [6] |
| Asp80 | x |  | x | 9 | Large changes in backbone angles [40].  Role in C-type inactivation in interaction with Trp68 [68].  Interacts with Glu71, may have influence in gating [9]. Influence gating [6] |
| Leu81 |  | x | x | 6 | C type inact. [21]. |
| Tyr82 |  | x | x | 1 | Large confor. Change, C type inact. [21,67].  Effect gating [9]. |
| Pro83 |  | x | x | 9 | Large conf. change, C type inact.  [21]. |
| Ala92 | x |  | x | 7 | Gating sensitive single mutations [11]. |
| Val93 | x |  |  | 5 | Gating sensitive single mutations [11]. |
| Val95 |  |  |  | 5 |  |
| Met96 |  |  | x | 4 | Gating sensitive single and double mutations (Thr75, Ala98) [11]. |
| Gly99 | x |  |  | 9 | Gly gating hinge [2]. |
| Ile100 | x |  |  | 8 | Gating sensitive double mutations (Thr75, Ser102) [11]. |
| Thr101 |  | x |  | 6 | Gating sensitive double mutations at Ser102 (28, Leu36, Thr75, Ala98, Ile100, Ala109) [11]. |
| Phe103 |  |  | x | 5 |  |
| Thr107 |  |  | x | 8 | Hinge by EPR study [5]. |
| Ala108 |  | x |  | 5 | Hinge by EPR study [5]. Hinge by computation [32] |
| Ala109 | x |  |  | 5 | Gating sensitive double mutations (Ala98) [11] |
| Leu110 | x |  |  | 7 |  |
| Ala111 | x | x |  | 5 |  |
| Thr112 | x |  |  | 7 |  |
| Trp113 | x |  |  | 5 |  |
| Phe114 | x |  | x | 9 | pH-dependent chemical shifts suggesting coupling wth the selectivity filter [6] |
| Gly116 | x | x |  | 5 | pH-dependent chemical shifts suggesting coupling wth the selectivity filter [6] |
| Arg117 | x |  | x | 8 |  |
| Glu118 |  |  | x | 5 |  |

^a^ The residues at which the sign of the cross-correlations changed in the analysis of single modes (1-through-7) by GNM.

^b^ The residues at which structural distortions accommodated the fluctuations observed in the two slowest modes by ANM (reflections from Figure 1).

^c^ The residues that interacted with the highest number of residues (at least seven residues with intra-subunit/ inter-subunit interactions) in the structure with an energy value above a threshold estimated by z-score analysis. See the caption of Figure 5.

^d^ Conservation grades by the *ConSeq* web-server [61]. The data was taken from Figure 4. The scale is from 1 to 9, where a grade of 1 signifies a highly variable amino acid position and 9- highly conserved.

^e^ Experimental data.
